# Genetic, histochemical and biochemical studies on goat TSE cases from Cyprus

**DOI:** 10.1186/s13567-016-0379-0

**Published:** 2016-10-06

**Authors:** Susanne Niedermeyer, Martin Eiden, Pavlos Toumazos, Penelope Papasavva-Stylianou, Ioannis Ioannou, Theodoros Sklaviadis, Cynthia Panagiotidis, Jan Langeveld, Alex Bossers, Thorsten Kuczius, Martin Kaatz, Martin H. Groschup, Christine Fast

**Affiliations:** 1 Institute of Novel and Emerging Infectious Diseases, Friedrich-Loeffler-Institute, Südufer 10, 17493 Greifswald-Isle of Riems, Germany; 2 Veterinary Services, Ministry of Agriculture, Rural Development and Environment, 1417 Nicosia, Cyprus; 3 Laboratory of Pharmacology, School of Pharmacy, Aristotle University of Thessaloniki, Thessaloniki, Greece; 4 Department of Infection Biology, Central Veterinary Institute of Wageningen UR, Lelystad, The Netherlands; 5 Institute for Hygiene, Westfälische Wilhelms-University and University Hospital Münster, Robert Koch-Strasse 41, 48149 Münster, Germany

## Abstract

Scrapie and bovine spongiform encephalopathy (BSE) are transmissible spongiform encephalopathies (TSE’s) affecting sheep and goats. Susceptibility of goats to scrapie is influenced by polymorphisms of the prion protein gene (PRNP) of the host. Five polymorphisms are associated with reduced susceptibility to TSE’s. In the study presented here caprine samples from a scrapie eradication program on Cyprus were genotyped and further characterized using BioRad TeSeE rapid test, histological, immunohistochemical and biochemical methods. In total 42 goats from 20 flocks were necropsied from which 25 goats showed a positive result in the rapid test, a spongiform encephalopathy and an accumulation of pathological prion protein (PrP^Sc^) in the obex. PrP^Sc^ deposits were demonstrated in the placenta, peripheral nervous and lymphoreticular system. Two animals showed PrP^Sc^-accumulations in peripheral tissues only. By discriminatory immunoblots a scrapie infection could be confirmed for all cases. Nevertheless, slight deviations in the glycosylation pattern might indicate the presence of different scrapie strains. Furthermore scrapie samples from goats in the current study demonstrated less long term resistance to proteinase K than ovine or caprine BSE control samples. Reduced scrapie susceptibility according to the PRNP genotype was demonstrated (Fishers Exact test, *p* < 0.05) for the goats with at least one polymorphism (*p* = 0.023) at the six codons examined and in particular for those with polymorphisms at codon 146 (*p* = 0.016). This work characterizes scrapie in goats having implications for breeding and surveillance strategies.

## Introduction

Transmissible spongiform encephalopathies (TSE’s) are progressive, fatal neurodegenerative diseases in humans and mammals. Causative is the conversion of the cellular membrane bound prion protein (PrP^c^) into a partially protease-resistant pathologic form (PrP^Sc^) [1]. TSE’s are characterized by long incubation periods of several months or even years. Among others, there is scrapie in sheep, goats and mufflons [2–4] and also bovine spongiform encephalopathy which was first described in cattle in 1986 [5] being the only animal prion disease confirmed to affect human beings (Creutzfeldt–Jakob disease) and mammals [6].

The first natural caprine scrapie case was not reported before 1942 [2], and, apart from diverse outbreaks in cattle, natural BSE infection was only diagnosed in one single British goat in 2005 [7]. Later on, a Scottish goat with BSE disease from 1990 was revealed in retrospective studies in the United Kingdom [8]. To date, natural BSE was never detected in sheep though the disease was successfully transmitted to sheep and goats under experimental conditions [9]. As already described for sheep, the susceptibility of goats for scrapie is highly influenced by polymorphisms of the prion protein gene (PRNP). To date, more than 40 polymorphisms of the caprine PRNP have been described in different countries and breeds [10–14]. Among these, only five polymorphisms were found which seem to correlate with TSE susceptibility. The polymorphisms I142M and R154H extend incubation period and for the latter partially protective effects had been described [12, 13, 15, 16]. Effects on resistance against TSE were associated with the polymorphisms N146S/D, R211Q and Q222K polymorphisms [12, 13, 17, 18].

As described for sheep, goats are typically diagnosed with scrapie at the age of 3 to 4 years [19], and clinical signs last from 2 weeks to 6 months [19–21]. Apathy, ataxia, severe pruritus, difficulties in milking and loss of weight are clinical signs seen in affected goats [20]. For sheep it is known that scrapie is transmitted horizontally under natural conditions via direct contact or from contaminated environment [22, 23]. The oral route is the most efficient entrance for the TSE material and the placenta after birth is the main source of infection [24–26]. However, PrP^Sc^ and/or infectivity have also been found in amniotic fluid [26], faeces [27], milk [28, 29] and even in the oral cavity of scrapie infected sheep [30]. Scrapie in goats is often found in mixed herds together with sheep [2, 31], but it has also been observed to spread from goat to goat [19].

Pathogenesis of scrapie has been widely described in sheep and to a lesser extent in goats. In sheep, first accumulations of PrP^Sc^ can be immunohistochemically detected in the tonsils and the gut associated lymphatic tissue such as the Peyers’ patches of the ileum and jejunum after oral ingestion [32–34].

Later on, PrP^Sc^ spreads and accumulates in the corresponding mesenteric lymph nodes [33, 35, 36] as well as in peripheral lymph nodes and the spleen [35, 37]. PrP^Sc^ deposits were also detected in the enteric nervous system with duodenum and ileum as first positive sites and with the tendency to spread extensively in the cranial and caudal parts of the ENS [32, 33]. It is widely accepted that PrP^Sc^ ascends along the parasympathetic and/or sympathetic nerve fibres with finally reaching the brain and the spinal cord, respectively [32]. As an additional pathway the hematogenous distribution of PrP^Sc^ is discussed considering PrP^Sc^ infectivity which was found in ovine blood [38] as well as the infection of the brain via circumventricular organs [39]. Studies describing natural scrapie in goats are rare but the data known so far indicate that the spread of PrP^Sc^ is comparable to classical scrapie in sheep [40–43]. However there can be a distinct variability among individual animals, which, among other things, might be due to a high variability of classical scrapie strains and their interactions with the particular genotype of the host [41].

Scrapie is spread nearly worldwide in sheep and goats [10, 31, 44, 45]. In Cyprus, scrapie has been a major problem for animal husbandry since the first detection in 1985 [31]. Thus within the European Union between 2007 and 2009, about 90% of the positive goat scrapie cases were found in Cyprus [41]. The applied extensive eradication programs were a unique possibility to collect samples from goats which were naturally infected with scrapie.

The study presented here describes a biochemical and immunohistochemical characterization of natural goat scrapie cases including PRNP genetics. It is aimed to shed more light on the pathogenesis of this disease in goats and also to characterize the diversity of natural scrapie strains on Cyprus more precisely excluding possible BSE cases at the same time.

## Materials and methods

### Animals

The goats were chosen within the framework of a scrapie eradication program that has been implemented in Cyprus. All animals were killed in accordance to the European animal welfare regulations. In total 42 Cypriot female goats of a local Damascus breed that were showing scrapie like clinical signs (alopecia, cachexia and/or ataxia) were sourced out from twenty flocks of the Nicosia district. Afterwards these goats of different ages were dissected in the facilities of the Veterinary Services (VS), Ministry of Agriculture, Rural Development and Environment, 1417 Nicosia, Cyprus. Beside blood samples a wide range of frozen and formalin fixed tissue samples were taken under TSE sterile conditions. All the 42 goats were kept together with sheep or were kept on pastures which formerly used sheep for grazing. Data of all goats are given in Tables 1 and 2.

**Table 1:** **Data and results of scrapie positive Damascus goats from Cyprus**

Goat ZYP	Flock (age)	Brain stem	CM GC	Ln. retroph.	Tonsil	3^rd^ eye lid	Spleen	Rectum		Placenta
		IHC (rapid test)						Follicles	ENS	
I_142_N_146_R_151_R_154_R_211_Q_222_										
3	B (4)	+++ (1.892)	+	NA	+++ 9/9	+++ 13/19	++ 83/202	+++ 1/1	neg.	neg.
8	C (4)	+++ (1.675)	NA	+++ 4/4	neg. 0/5	+++ 7/12	+ 6/140	neg. 0/3	+ Pl.my.	NA
9	D (4)	++ (1.579)	++	+++ 625/830	+++ 96/120	+++ 35/64	+++ 110/162	+++ 19/24	neg.	neg.
10	E (3)	+++ (1.985)	NA	+++ 232/646	++ 124/213	+++ 5/18	+ 93/428	+++ 37/48	+ Pl.my.	neg.
11	F (4)	+++ (1.856)	+	+ 93/284	+++ 124/255	neg. 0/18	+ 10/374	neg. 0/19	+ Pl.my.	neg.
12	G (3)	+++ (2.193)	++	++ 190/502	0	+ 9/46	+ 126/396	++ 71/152	neg.	neg.
13	G (4)	+++ (1.992)	+++	+ 87/365	+++ 111/145	+++ 4/4	+++ 56/99	+++ 131/174	++ Pl.my/+sub.	NA
14	F (4)	+++ (1.871)	+++	+++ 77/139	++ 174/307	+++ 5/5	+++ 167/246	+++ 28/46	++ Pl.my/+sub.	neg.
16	H (4)	+++ (2.204)	+++	+ 147/363	+ 20/78	+ 1/10	+ 64/270	++ 19/47	+ Pl.my/+sub.	NA
17	H (4)	+++ (2.107)	++	++ 119/300	+++ 72/132	+++ 15/17	+ 65/160	+++ 29/43	+ Pl.my.	NA
19	H (4)	++ (1.831)	+	++ 50/131	+ 15/82	+ 1/9	+++ 148/234	+++ 2/2	++ Pl.my.	NA
20	I (3)	+++ (1.961)	+	++ 18/57	+++ 268/335	+ 2/7	++ 123/373	+++ 26/43	++ Pl.my/+sub.	neg.
21	J (5)	++ (1.511)	+++	+ 190/615	NA	+ 4/41	++ 175/314	+ 20/37	+++ Pl.my/+sub.	+
22	J (5)	++ (1.151)	NA	NA	+++ 65/96	neg. 0/2	+ 42/269	+ 1/41	+ Pl.my.	NA
25	K (3)	++ (1.227)	++	+ 278/654	+++ 376/585	+++ 3/4	++ 173/339	++ 4/14	++ Pl.my/+sub.	NA
26	L (4)	++ (1.731)	+	+++ 196/302	0	neg. 0/26	+ 133/571	+ 55/146	+++ Pl.sub.	+
27	M (3)	++ (1.141)	+	+++ 229/344	+++ 41/65	+++ 17/23	+++ 82/152	++ 10/28	+++ Pl.my/+sub.	+
29	M (4)	++ (1.921)	++	+++ 180/313	+ 290/459	+++ 67/85	+++ 125/128	+++ 21/37	++ Pl.my/+sub.	neg.
30	M (6)	+++ (1.246)	++	++ 90/286	+++ 72/139	neg. 0/2	+ 81/224	+++ 17/22	+ Pl.my/+sub.	neg.
31	N (4)	++ (1.255)	++	+++ 264/398	+++ 131/144	+++ 26/55	++ 169/366	+++ 66/92	+++ Pl.my/+sub.	neg.
33	O (2)	++ (1.784)	++	+++ 136/262	+++ 141/164	++ 3/10	++ 257/503	+++ 30/39	++ Pl.my/+sub.	NA
34	P (2)	++ (1.376)	+++	+++ 626/878	+++ 191/256	+++ 8/9	+++ 228/292	+++ 6/7	+ Pl.my.	NA
35	P (4)	+++ (1.452)	NA	+++ 319/625	+++ 73/147	0	+ 4/208	+ 9/101	+ Pl.my.	neg.
41	O (4)	++ (1.674)	NA	++ 97/268	+++ 72/102	neg. 0/14	+ 60/167	0	+ Pl.my.	NA
24	K (2)	neg. (0.012)	+	+ 391/1154	NA	neg. 0/42	+ 11/446	+ 8/80	neg.	NA
37	Q (3)	neg. (0.010)	+	neg. 0/168	neg. 0/73	neg. 0/8	neg. 0/149	neg 0/14	neg.	NA
R/H154										
40	R (4)	++ (1.069)	+	+ 21/220	neg. 0/6	neg. 0/6	neg. 0/134	neg. 0/35	+++ Pl.my.	neg.

D: aspartic acid; I: isoleucine; H: histidine; M: methionine; N: asparagine; Q: glutamine; R: arginine; S: serine; A–T: goat herds; age: is given in years; Cut-off BioRad rapid test: 0.213; IHC: immunohistochemistry; +, ++, +++: mild, moderate, severe PrP^Sc^ accumulation (the first number indicates the number of positive follicles, the second number the total number of follicles examined); CMGC: celiac and mesenteric ganglion complex; Ln. retroph.: retropharyngeal lymph node; 0: no lymphatic follicles; ENS: enteric nervous system; Pl. my.+sub.: myenteric/submucosal plexus; neg.: PrP^Sc^ negative; NA: tissue sample not available.

**Table 2:** **Data and results of all scrapie negative Damascus goats from Cyprus**

Goat ZYP	Flock (age)	Brain stem	CM GC	Ln. retroph.	Tonsil	3^rd^ eye lid	Spleen	Rectum		Placenta
		IHC (rapid test)						Follicles	ENS	
I_142_N_146_R_151_R_154_R_211_Q_222_										
6	A (7)	neg. (0.014)	neg.	neg. 0/120	neg. 0/45	neg. 0/13	neg. 0/231	NA	NA	NA
18	H (4)	neg. (0.016)	neg.	neg. 0/97	neg. 0/111	neg. 0/21	neg. 0/3741	neg. 0/9	neg.	NA
23	K (6)	neg. (0.011)	neg.	neg. 0/110	neg. 0/64	neg. 0/3	neg. 0/201	neg. 0/116	neg.	NA
32	O (4)	neg. (0.018)	neg.	neg. 0/401	0	neg. 0/9	neg. 0/173	neg. 0/47	neg.	NA
38	Q (3)	neg. (0.010)	neg.	neg. 0/217	neg. 0/186	0	neg. 0/276	neg. 0/49	neg.	NA
42	S (2)	neg. (0.007)	neg.	neg. 0/388	neg. 0/149	neg. 0/1	neg. 0/345	neg. 0/5	neg.	neg.
43	S (3)	neg. (0.008)	neg.	neg. 0/98	neg. 0/2	neg. 0/39	neg. 0/313	neg. 0/35	neg.	neg.
44	S (4)	neg. (0.008)	neg.	neg. 0/329	neg. 0/73	neg. 0/26	neg. 0/306	neg. 0/46	neg.	NA
45	T (5)	neg. (0.010)	neg.	neg. 0/359	neg. 0/132	neg. 0/4	neg. 0/223	neg. 0/16	neg.	NA
46	T (4)	neg. (0.010)	neg.	neg. 0/548	neg. 0/195	neg. 0/51	neg. 0/294	neg. 0/14	neg.	NA
N/S146										
2	A (7)	neg. (0.017)	neg.	neg. 0/219	neg. 0/86	neg. 0/1	neg. 0/267	neg. 0/1	neg.	NA
S146										
4	A (7)	neg. (0.049)	neg.	neg. 0/230	neg. 0/158	0	neg. 0/106	neg. 0/2	neg.	NA
N/D146										
28	M (2)	neg. (0.010)	neg.	neg. 0/223	neg. 0/204	neg. 0/18	neg. 0/207	neg. 0/31	neg.	NA
M142										
36	Q (3)	neg. (0.010)	neg.	neg. 0/131	neg. 0/49	neg. 0/3	neg. 0/270	neg. 0/2	neg.	NA
N/D146R/H151										
39	Q (4)	neg. (0.007)	neg.	neg. 0/899	neg. 0/2	neg. 0/21	neg. 0/168	neg. 0/3	neg.	neg.

D: aspartic acid; I: isoleucine; H: histidine; M: methionine; N: asparagine; Q: glutamine; R: arginine; S: serine; A–T: goat herds; age is given in years; Cut-off BioRad rapid test: 0.213; IHC: immunohistochemistry; +, ++, +++: mild, moderate, severe PrP^Sc^ accumulation (the first number indicates the number of positive follicles, the second number the total number of follicles examined); CMGC: celiac and mesenteric ganglion complex; Ln. retroph.: retropharyngeal lymph node; 0: no lymphatic follicles; ENS: enteric nervous system; neg.: PrP^Sc^ negative; NA tissue sample not available.

### Genotyping

The genotyping of the codons 142, 146, 151, 154, 211 and 222 of the prion protein gene (PRNP) was done by C. H. Panagiotidis at the Aristotle University in Thessaloniki Greece using the cycle-sequencing method formerly described [46].

### Tissue samples

**Figure 1:**
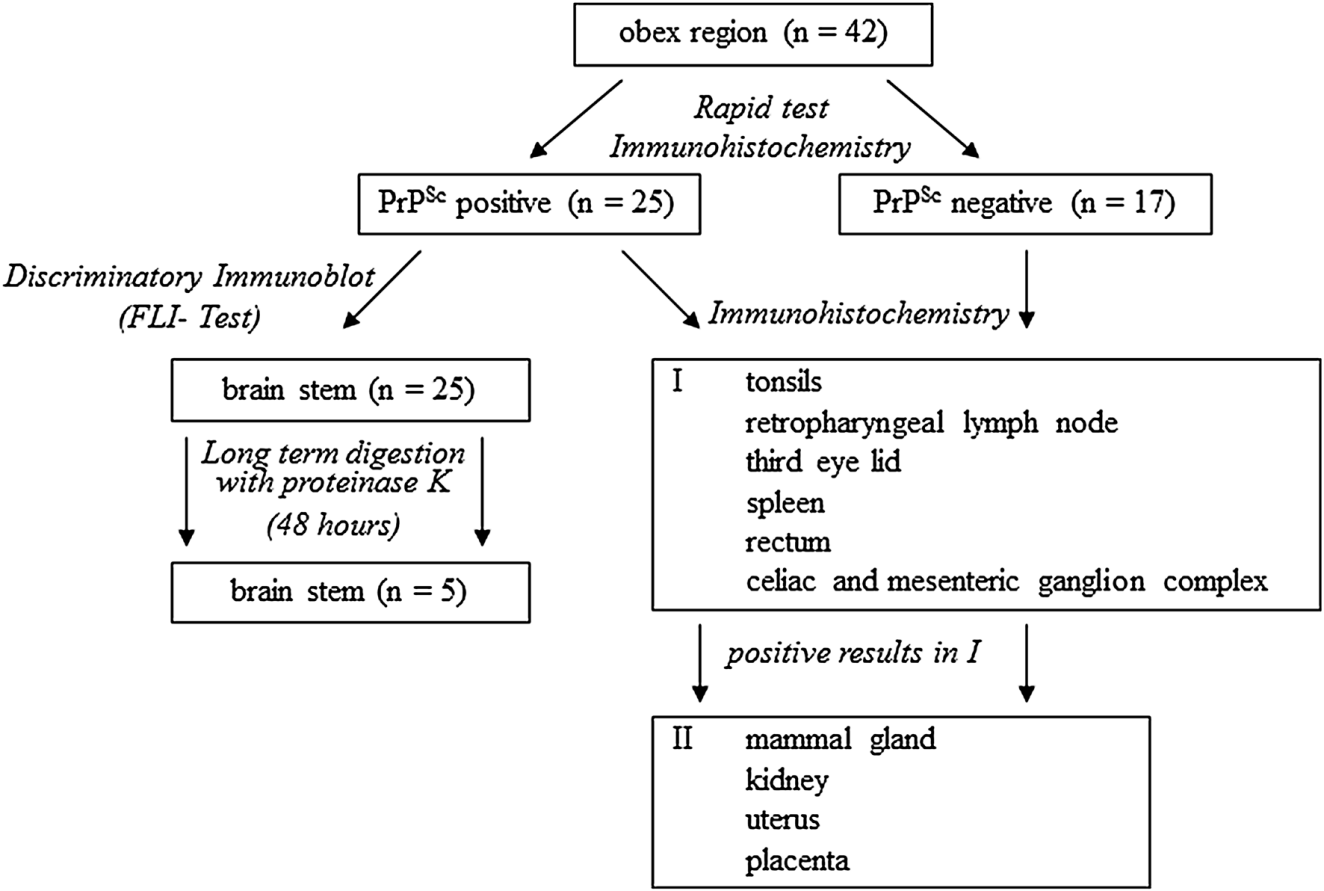
**Schema of immunohistochemical and biochemical tests carried out on selected samples of the Cypriot goats.** At first, brain stem samples of all 42 goats were examined by different methods. Subsequently the most likely sites of PrP^Sc^ accumulations such as the retropharyngeal lymph node, tonsils, third eye lid, spleen, celiac and mesenteric ganglion complex (CMGC) and rectum of all goats were examined by immunohistochemistry (I). In a second step, samples of the kidney, mammary gland, uterus and, if available, placentomal tissue of the placenta were examined with only taking the goats into account which revealed PrP^Sc^ positive results in the tissue samples analysed previously (II). Additionally all positive brain stem samples were examined by discriminatory immunoblot.

Selected tissue samples were examined following different analysis procedures (Figure 1). Brain stem material (obex region) of all 42 goats was examined histologically, immunohistochemically and biochemically. Further samples were examined immunohistochemically only. In this case, to limit the samples sizes which had to be examined, the most likely sites of PrP^Sc^ accumulations such as the retropharyngeal lymph node, tonsils, third eye lid, spleen, celiac and mesenteric ganglion complex (CMGC) and rectum of all goats were examined in a first step. In a second step, samples of the kidney, mammary gland, uterus and, if available, placentomal tissue of the placenta were examined with only taking the goats into account which revealed PrP^Sc^ positive results in the tissue samples analysed previously.

### Histology and immunohistochemistry

The tissue samples as well as the resulting sections were treated as described before [47] with following modifications. Sections were cut in serials by producing sections of up to three areas per paraffin block. The three areas were chosen in a 25–30 µm distance what guaranteed a totally different cell layer in each area examined. Sections of all samples were stained with haematoxylin and eosin. For the detection of caprine PrP^Sc^, the three different PrP-specific monoclonal antibodies (mAb) 6C2 (Central Veterinary Institute of Wageningen UR, Lelystad, Netherlands), F99 (VMRD, Pullman, USA) and L42 [48] which were raised to the epitope 114–120 of bovine PrP protein (6C2) and to epitop 220–225 (F99) and epitop 145–163 (L42) of ovine PrP protein, respectively, were chosen. Before applying the antibodies, the sections had been pre-treated by incubation for 30 min (6C2, F99) or 15 min (L42) in 98% formic acid and rinsed in tap water for 5 min. After inhibiting the endogenous Peroxidase the sections had been autoclaved in citrate buffer for 20 min at 121 °C and 3 bar (6C2, F99) or pre-treated by proteinase K (PK, 4 µg/mL) digestion (L42). The primary antibodies were applied at a dilution of 1:50 up to 1:150 (6C2) depending on the stock concentration and 1:10 000 (F99) or 1:250 (L42) in goat serum including 0.03% sodium azide and incubated for 2 h at room temperature. The process was finished as described by [47].

### Rapid testing and biochemical methods

Brain stem material of all necropsied goats was primarily analysed in the TSE Section of the Laboratory of Animal Health (LAH) of VS by applying the BioRad TeSeE (München, Germany) rapid test in line with the manual instructions. Optical density (OD) values more than twofold above the cut-off were defined as clear reactive samples.

The identified TSE-positive samples and BSE and scrapie references were additionally analyzed in the discriminatory immunoblot, the so called FLI-test. The latter included a phosphotungic acid (PTA) precipitation of PrP^Sc^ followed by SDS-PAGE and immunoblotting, as described before [49] with the following modifications: In general, a 100 µL-aliquot of brain homogenates was subjected to precipitation. However, depending on the content of PrP^Sc^ and the resulting signal intensity obtained from the immunoblot, the volume of brain homogenate varied from 30 µL up to 200 µL. A second precipitation step was included to optimize the original PTA precipitation protocol. First, the resulting pellet was homogenized in 45 µL of 50 mM Citrate buffer (pH 6.0/NaOH) containing 200 mM KCl, 5.0 mM MgCl_2_ and 1.25% (w/v) sarcosine. A volume of 0.5 µL of thyroglobulin, suspended in bi-distilled water (5 mg/mL), was added followed by vortexing in 500 µL of methanol (99.8%) and storage at −20 °C for at least 1 h. After centrifugation for 30 min, the supernatant was removed and the pellet was dried under constant shaking at 37 °C. All centrifugation steps were carried out at 17 000 *g*. Finally the pellet was suspended in a sample buffer for SDS-PAGE as described before [51]. The two antibodies mAb L42 and the mAb P4 (R-Biopharm) were selected. As standard marker the FLI-marker was used [50]. Banding patterns were revealed using the chemiluminescence substrate CDP-Star (Tropix, USA). After incubation for 5 min, emitted light signals were recorded by a photo imager system (Versadoc, Bio-Rad, Germany). Positive controls such as classical ovine scrapie and bovine BSE brain material were included in each assay. Molecular masses were assessed by concurrent FLI-marker [50] and determined by the analysis software Quantity One (BioRad, Germany). The values were calculated as mean ± SD of at least four independent determinations.

The FLI-test identifies the different cleavage sites of the PK for BSE and scrapie which are varying between the amino acids 96 to 97 and between the amino acids 81 to 89, respectively. Thus, the persisting PrP^Sc^ fragments of BSE are smaller than those of the most scrapie PrP^Sc^ fragments after the PK digestion. To discriminate BSE from scrapie three parameters have to be met as described elsewhere [49]. The molecular weight of the un-glycosylated form of the PrP^Sc^ has to be compared with the un-glycosylated form of the PrP^Sc^ of a positive scrapie control. A BSE isolate shows a lower molecular weight than the scrapie control (of >0.5 kDa). In the immunoblot, the PrP^Sc^ fragments are visualized using antibody binding sites either at the PrP core region (mAb L42) or at an N-terminal site, which is still present in PK treated PrP^Sc^ (mAb P4) of scrapie, but removed in that of BSE. Therefore the mAb P4 binds to the PrP^Sc^ of BSE only weakly or does not bind at all, which results in a P4/L42 binding ratio of <0.4. The epitope specificities of these antibodies have been shown earlier [51]. Finally, following the glycosylation pattern of the PrP^Sc^, samples are BSE-like when showing a percentage of the diglycosylated form of the total PrP^Sc^ signal of >50.0%.

### Analysis of long-term proteinase K (PK) resistance

Long term resistance against proteolysis with proteinase K was determined for five selected isolates after 48 h exposure. A 600 µL-aliquot of equally homogenized brain material, diluted with phosphate buffered saline (PBS) to a final concentration of 20 mM, was adjusted with magnesium chloride to a final concentration of 1 mM and with benzonase to a final concentration of 50 U/mL and incubated with PK (final concentration 100 µg/mL) at 55 °C and under continuous shaking. After 1, 6, 24 and 48 h of proteolysis, 150 µL each was taken for PTA precipitation of PrP^Sc^, which was done without heat denaturizing at 95 °C for 5 min, followed by SDS-PAGE and immunoblotting and photo imaging as for the FLI-test [49]. The test to determine long term PK resistance was repeated four times for each sample and data were given as the mean of the four independent determinations.

### Controls, references and comparative samples

Control and positive reference material for immunohistochemical and biochemical examinations was taken from ovine, caprine or bovine brain stem samples which had been tested scrapie and BSE-negative or scrapie and BSE-positive, respectively, from the national reference laboratory for transmissible spongiform encephalopathy of the Friedrich–Loeffler Institute (FLI), Isle of Riems, Germany. As comparative samples for biochemical examinations were additionally used BSE-positive brain stem materials, experimental generated in sheep (486; Frederic Lantier, INRA, France) and goat (ZG01; FLI, Germany) and one single scrapie-positive (Stdl. 171) as well as one single bovine sample (R 8/09), which had been submitted for diagnostic purposes to the FLI before.

### Statistical analysis

The statistical analysis was done using the exact Fisher test taking into account a maximum error probability of 5% (α = 5%). Consequently, differences [between the results (p)] were only labeled statistically significant with a *p* smaller 0.05 (*p* < 0.05). Statistical calculation was done using R version 2.8.1 (2008).

## Results

The 42 goats which were sourced from the different herds due to scrapie-like clinical signs like alopecia, cachexia and ataxia were tested by BioRad TeSeE rapid test immediately after necropsy. Twenty-five of the samples were tested PrP^Sc^ reactive with OD values between 1.069 and 2.204 (cut-off = 0.213) and 17 samples showed clearly negative results with OD values between 0.007 and 0.049 (cut off = 0.213). The rapid test results were confirmed by immunohistochemistry using mAb 6C2.

The TSE positive goats exhibited the I_142_N_146_R_151_R_154_R_211_Q_222_ genotype (wild-type) with one exception (goat ZYP40) that showed a polymorphism at codon 154 (R154H). The goats were two (*n* = 2), three (*n* = 5), five (*n* = 2), six years (*n* = 1) and most of them four years old (*n* = 15). The variability of genotypes was higher among the TSE negative goats, exhibiting in four animals single polymorphisms as N146S (*n* = 1), S146S (*n* = 1), N146D (*n* = 1) and M142 M (*n* = 1), respectively. In addition, one goat revealed a N146D and a R151H polymorphism at the same time. Again, most of the TSE negative goats were four years old (*n* = 5), and four goats had been 3 years old. The remaining TSE negative goats were two (*n* = 3), five to six (*n* = 1 each) or 7 years old (*n* = 3), which points to a greater number of the younger and the older goats. The compilation of all data can be seen in Tables 1 and 2.

### Biochemical characterization

**Figure 2:**
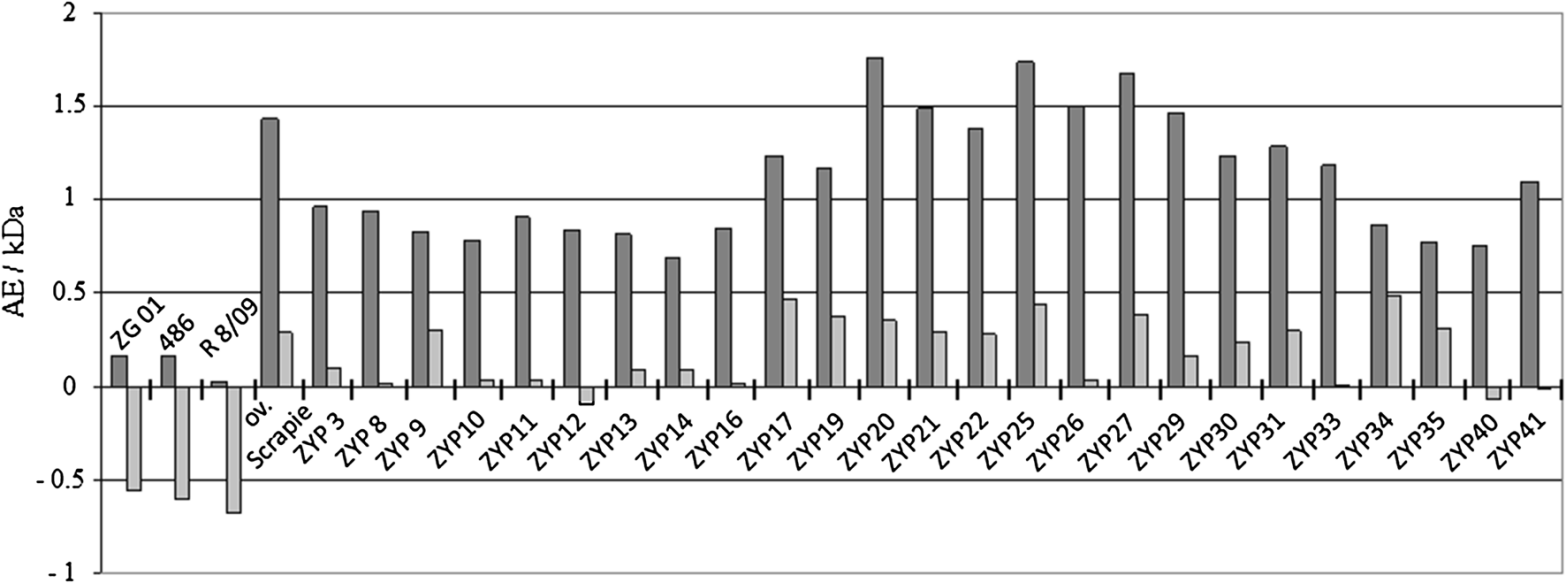
**Discriminatory immunoblot of goats with PrP** ^**Sc**^ **accumulation in the brain stem.** Antibody-binding ratios P4/L42 and the differences of the molecular weights of proteinase K treated, unglycosylated PrP^Sc^ bands/form in contrast to the molecular weight of the sheep scrapie control are shown of bovine (R8/09), ovine (486) and caprine (ZG01) BSE, of ovine scrapie (Stdl. 171) and all Cypriot caprine scrapie samples. P4/L42 ratios were calculated as the intensity of the signal strength from P4 versus L42 gained from Western blot.  Y-axis: dark grey, P4/L42 antibody binding ratio (AE = arbitrary unit), data obtained by two gel runs;  light grey, difference molecular weight of the un-glycosylated form of the PrP^Sc^ sample and scrapie control (kDa), data obtained by four to five gel runs.

Brain stem samples of the 25 as PrP^Sc^ positive identified goats were further characterized using the FLI test. Therein, the glycosylation pattern, the percentage of the di-glycosylated form of the PrP^Sc^ and the molecular weight of the non-glycosylated form of the PrP^Sc^ were determined for each. In addition, from five selected samples (from the goats ZYP13, ZYP19, ZYP21, ZYP27 and ZYP30) the long term resistance against PK proteolysis was determined over 48 h. For comparative purposes, a caprine (ZG01), an ovine (486) and a bovine BSE sample (R 8/09) and an ovine classical scrapie isolate (Stdl. 171) were characterized in the same manner. The molecular weight of the non-glycosylated form of PrP^Sc^ was determined to be between 18.0 and 19.4 kDa for the Cypriot caprine scrapie isolates. The ovine scrapie isolate and the caprine, ovine and bovine BSE isolates exhibited molecular weights of 19.2 kDa, 18.8 kDa, 18.7 kDa and 18.5 kDa, respectively. Referred to the mean of the four to five independent determinations of the molecular weight, only a part of the caprine scrapie isolates (*n* = 12) could be differentiated from the caprine, ovine and bovine BSE isolates. However, all of the Cypriot samples showed a molecular weight of the non-glycosylated form of the PrP^Sc^ clearly above the internal BSE reference of each blot. The difference of the molecular weight of the non-glycosylated form of the PrP^Sc^ between the Cypriot isolates and the scrapie reference did not exceed 0.5 kDa (−0.1 to 0.49 kDa). The ovine scrapie isolate (Stdl. 171) showed a comparable result (0.29 kDa), whereas molecular weights of the three BSE isolates were more than 0.5 kDa smaller than those of the internal scrapie reference (−0.67 to −0.56 kDa) (Figure 2).

The P4/L42 antibody binding ratio of the Cypriot isolates ranged between 0.69 and 1.76 and thus indicated a clear scrapie-like parameter. In a comparable manner, the ovine scrapie isolate (Stdl. 171) showed an antibody binding ratio of 1.43. For the caprine, ovine and bovine BSE isolates, however, distinct different ratio values of 0.16, 0.16 and 0.03 were determined (Figure 2).

**Figure 3:**
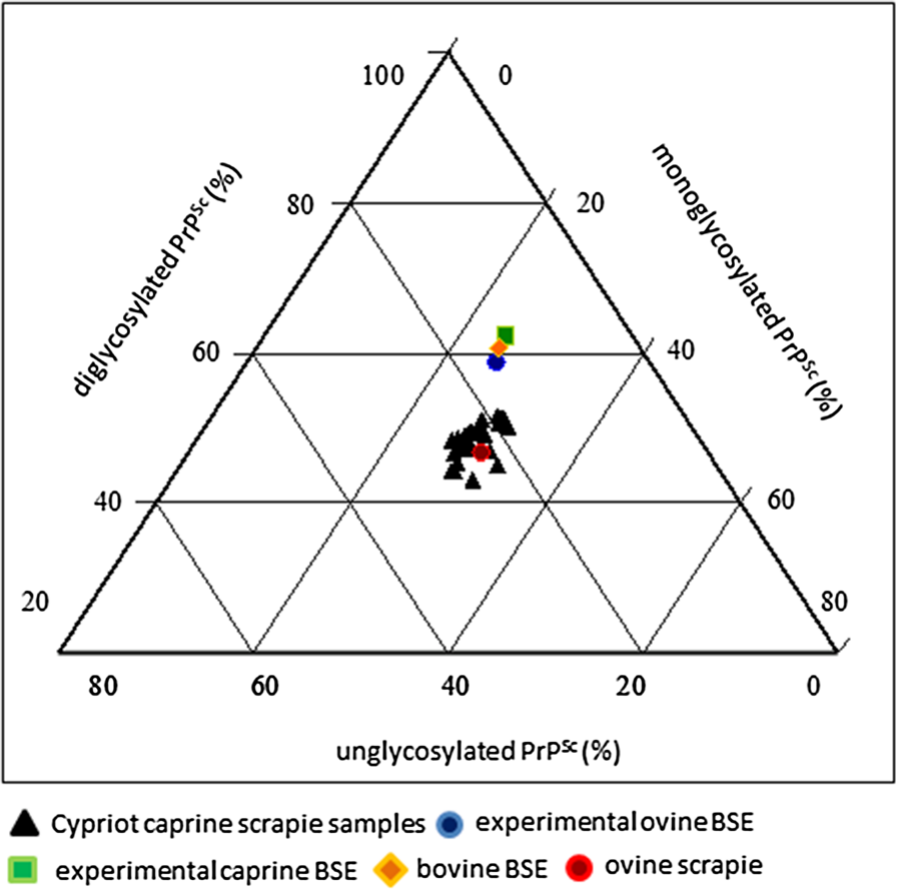
**Discriminatory immunoblot of goats with PrP** ^**Sc**^ **accumulation in the brain stem.** Glycosylation patterns of the 25 caprine isolates from Cyprus, the caprine (ZG01), ovine (486) and bovine (R8/07) BSE isolates and of the ovine scrapie isolate (Stdl. 171) are shown in a comparative tri-plot diagram. On the left axis is demonstrated the proportion of the di-glycosylated, on the right axis of the mono-glycosylated and on the axis below the un-glycosylated form of PrP^Sc^ of the overall signal in the western blot. The Cypriot samples as well as the ovine scrapie case can be clearly differentiated from the BSE samples.

The ratio of the di-glycosylated to the mono-glycosylated form of the PrP^Sc^ ranged between 43 and 52%:26 and 32% for the goat samples, meanwhile for the ovine scrapie sample the ratio was 46:30%. In contrast, the BSE isolates showed a more dominant di-glycosylated form of the PrP^Sc^ with ratios of 62:25% for the caprine, 59:26% for the ovine and 61:25% for the bovine BSE sample, respectively (Figure 3). Interestingly, eight of the caprine scrapie isolates showed a percentage of the di-glycosylated form of the PrP^Sc^ of >50% (ID ZYP13, ZYP17, ZYP19, ZYP25, ZYP27, ZYP29, ZYP30 and ZYP33).

In sum, applying the FLI-test, the Cypriot caprine isolates as well as the ovine scrapie isolate (Stdl. 171) clearly showed scrapie-like biochemical characteristics and BSE could be excluded for the former isolates without any exception. Though eight samples showed a portion of the di-glycosylated form of the PrP^Sc^ higher than 50%, which points to BSE-like characteristics, the other two parameters (difference of molecular weight of the non-glycosylated form of the PrP^Sc^ to the internal scrapie reference and P4/L42 antibody binding ratio) clearly ascertain scrapie for these isolates.

Those goats, showing a percentage of the di-glycosylated form of the PrP^Sc^ higher than 50.0%, all of them belonged to the I_142_N_146_R_151_R_154_R_211_Q_222_ wild type genotype coming from neighboring herds. At the same time, goats from the same (herds G, H, K, M and O) and neighboring herds (e.g. herds F and P) were tested scrapie positive showing clearly scrapie-like characteristics for this parameter (Table 1).

Nevertheless, BSE-like characteristics were verified for all control BSE samples of goat, sheep and cattle (ZG 01, 468 and R 8/09), which all fulfil the three criteria of the FLI-test [49].

**Figure 4:**
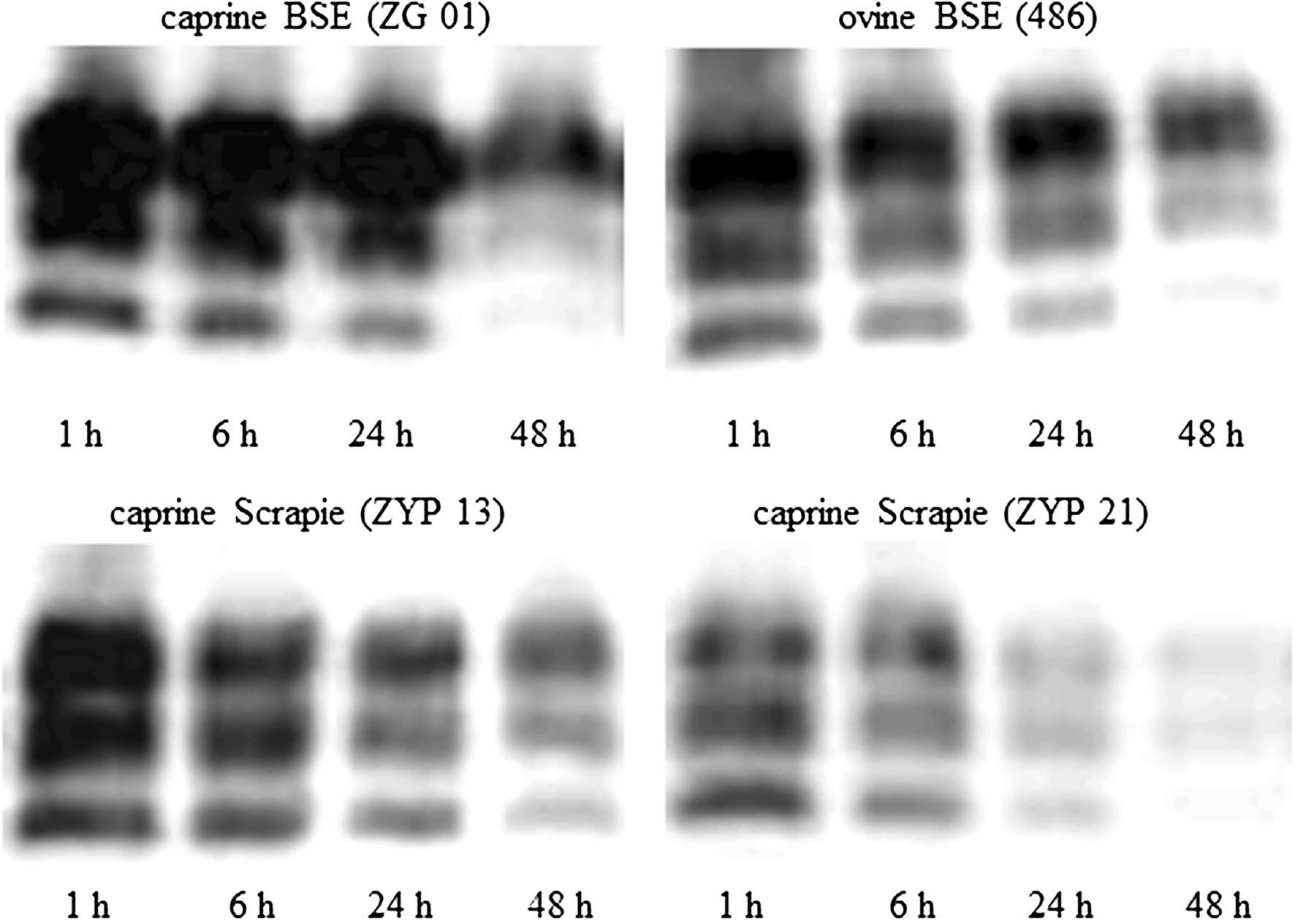
**Western blot showing PK resistence of PrP** ^**Sc**^ **from Cypriot goats as compared to BSE controls.** Resistance of PrP^Sc^ of caprine (ZG01) and ovine BSE (486) isolates as well as two Cypriot caprine scrapie isolates (ZYP13, ZYP21) after digestion with proteinase K over 1, 6, 24 and 48 h and visualization in the Western blot using antibody L42.

**Figure 5:**
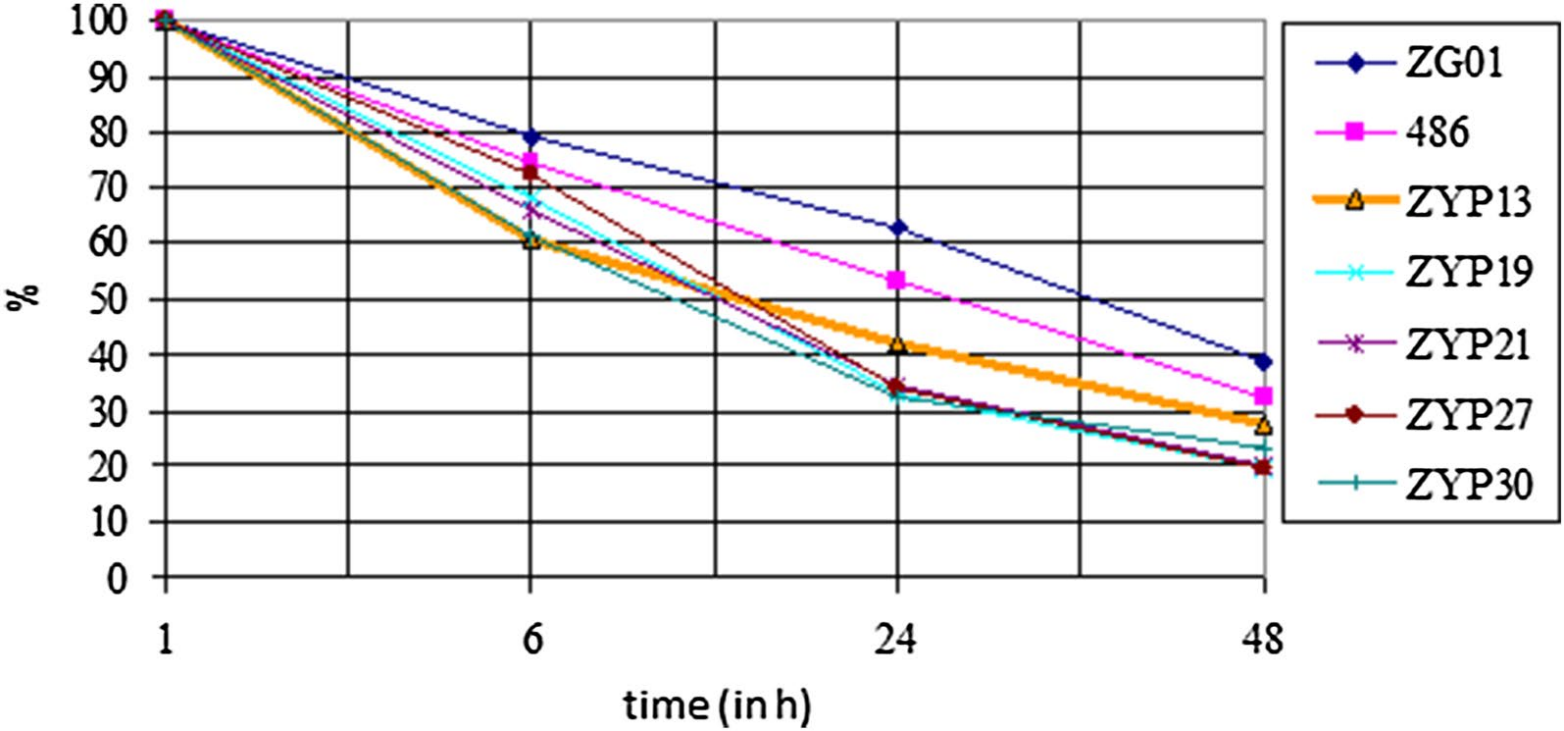
**Diagram showing PK resistance of PrP** ^**Sc**^ **from Cypriot goats as compared to BSE controls.** Proteinase K-resistance of PrP^Sc^ after 1, 6, 24 and 48 h digestion, showing the higher susceptibility of the different scrapie cases (ZYP13, ZYP19, ZYP21, ZYP27, ZYP30) compared to ovine (486) and caprine BSE (ZG01). The signal of the PrP^Sc^ in the western blot is shown as a percentage of the overall signal after 1 h (=100%). The data are obtained by four gel runs (ZYP21 = three gel runs).

In addition, the PK resistance was determined for five selected Cypriot goat samples and the ovine and caprine BSE samples. Four of the selected samples revealed in the FLI-test a glycosylation pattern with >50.0% of the diglycosylated form of the PrP^Sc^ and the one remaining sample (ZYP21) showed typical scrapie-like parameters in the FLI-test as exhibited by most of the Cypriot scrapie isolates. All the five Cypriot scrapie isolates (ZYP13, ZYP19, ZYP21, ZYP27 and ZYP30) tended to a lower proteinase K (PK) resistance over a 48 h period than the ovine (486) and caprine (ZG01) BSE reference samples (Figure 4). After 6, 24 and 48 h, 61–72%, 33–42% and 20–28% of the PrP^Sc^ of the scrapie isolates remained undigested compared to 75–79%, 53–63% and 33–39% of the non-digested BSE isolates (Figure 5). Thus the caprine BSE isolate was slightly more resistant against PK digestion.

**Figure 6:**
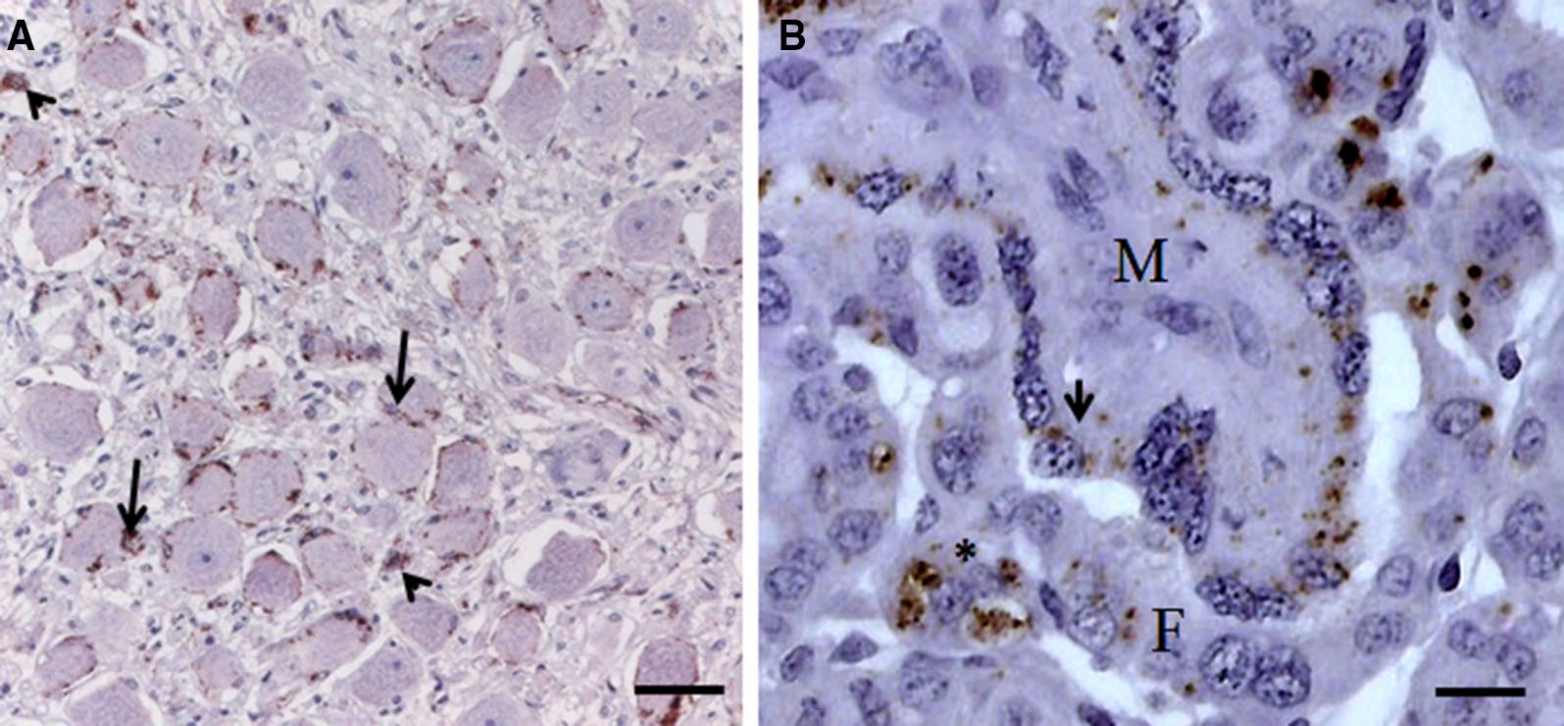
**Immunohistochemical detection of PrP** ^**Sc**^ **in autonomic nervous system and placenta. A** Celiac and mesenteric ganglion complex of a scrapie infected goat (ZYP30) with moderate accumulation of PrP^Sc^. Besides a perineuronal staining reaction, intracellular PrP^Sc^ deposits can be seen in associated satellite cells (arrows) as well as in multifocal glial cells (arrowheads); mAb 6C2; bar 20 µm; **B** Mild PrP^Sc^ accumulations in trophoblasts (*) and epithelial cells (arrow) in placental cotyledons of goat ZYP27. M: maternal parts; F: fetal parts; mAb 6C2, bar 10 µm.

### Histological and immunohistochemical results

All the 25 goats with a positive reactive Biorad TeSeE rapid test result exhibited in the obex region distinct signs of a spongiform encephalopathy and a clear PrP^Sc^ accumulation. For histological and immunohistochemical examinations six regions of the obex region like the dorsal motor nucleus of the vagus nerve (DMNV), the nucleus of the solitary tract, cuneate nucleus, hypoglossal nucleus, spinal tract nucleus of the trigeminal nerve and olivary nuclei were evaluated. Nine of the animals showed mild histopathological alterations which were mainly confined to the DMNV, the nucleus of the solitary tract and most prominent in the spinal tract nucleus of the trigeminal nerve. Nine goats were affected moderately and seven showed severe histological spongiform alterations in most of the brain stem nuclei. Referring to all samples, the olivary nuclei were affected only sporadically.

About half of the samples (*n* = 13) revealed moderate deposits of PrP^Sc^ with prominent immunoreactivity in the DMNV, whereas the other half (*n* = 12) was severely affected by PrP^Sc^ depositions. The hypoglossal nucleus, however, was less involved in this process. Using C-terminal antibodies, intra-neuronal, intra-glial and cell-membrane associated/extra-cellular fine up to coarse PrP^Sc^ accumulations were detected.

All 25 scrapie-positive animals showed depositions of PrP^Sc^ at least in one sample of the LRS and/or the celiac and mesenteric ganglion complex (CMGC), mostly in a moderate up to a severe degree (Figure 6). Two additional goats (ZYP37 and ZYP24)—with no detectable PrP^Sc^ in the brain stem—showed positive staining reactions in peripheral samples, only. In both goats mild PrP^Sc^ accumulations were detected in the CMGC. ZYP24 showed additional PrP^Sc^ deposits in different samples of the LRS. Overall, PrP^Sc^ accumulations were of a clear lesser degree as those which were found in the samples of the CMGC and LRS of the goats with PrP^Sc^ positive brain stem samples. Not for all goats a complete set of samples was available. Moreover in some LRS and CMGC samples follicles or neurons for the evaluation were missing. An overview concerning the immunohistochemical findings in the peripheral tissues is given in Tables 1 and 2.

The retropharyngeal lymph node revealed the most frequent PrP^Sc^ detection in the LRS (24/24), followed by spleen (24/25), tonsils (18/20), lymphoid follicles of the rectum (22/25) and the nictitating membrane (18/26). Intracellular fine to coarse PrP^Sc^ deposits were detected in tingible body macrophages and lymphocytes as well as in follicular dendritic cells of the LRS. The CMGC showed also frequent mild up to severe PrP^Sc^ detection (22/22) with a peri- and intraneuronal staining reaction as well as an intracellular accumulation in associated satellite cells. Multifocally distributed glial cells showed a PrP^Sc^ deposition, too (Figure 5A). Intraaxonal PrP^Sc^ depositions were seen rarely.

Furthermore, PrP^Sc^ accumulations were also detected in the ENS of the rectum (22/26), mostly associated with additional depositions in lymphoid follicles. Neurons and glial cells of both plexus were affected, but more frequently in the myenteric plexus (21/22) as compared to the submucosal plexus (12/22). Additionally placental tissue samples from fifteen ewes were analyzed. In doing so, rare intracellular PrP^Sc^ depositions were demonstrated in trophoblasts and epithelial cells of the cotyledons from the goats ZYP21, ZYP26 and ZYP27 (Figure 6). No PrP^Sc^ accumulation was detectable in kidney (0/24), mammary gland (0/23) or uterus (0/23).

## Discussion

In this study 42 goats from 20 different flocks showed clinical signs suspicious for classical scrapie and were culled within the context of a scrapie eradication program in Cyprus. The animals were sourced out by local veterinarians due to unspecific clinical signs which might be indicative for scrapie like alopecia, cachexia and ataxia. However, these signs are not specific for scrapie and there was no possibility for detailed clinical examinations, which explains the comparable high number of negative cases among the animals included in the study. Age distribution of the TSE positive goats was heterogeneous from two up to 6 years of age (mean 3.8 years), which is in accordance with previous reports of classical scrapie in goats [12, 20, 52]. The goats were analysed to get data on genotypic impact on scrapie susceptibility, pathologic pathways of caprine scrapie, and finally to differentiate it from BSE infection applying discriminatory tests.

Twenty-five of these goats were tested scrapie positive and 17 scrapie negative, based on findings in the brain stem samples. In this latter group two goats showed PrP^Sc^ deposits in peripheral sites only. Most of the 25 scrapie positive goats belonged to the I_142_N_146_R_151_R_154_R_211_Q_222_ wild type genotype and had widespread PrP^Sc^ accumulations in samples of the CMGC, in the LRS and in the rectal ENS. As an exception, one single goat (ZYP40) had peripheral PrP^Sc^ depositions, which were restricted to the CMGC (mild accumulations), the retropharyngeal lymph node and to the rectal ENS (severe accumulations). This goat had a R/H polymorphism on codon 154. Additionally two of the 17 scrapie rapid test negative goats had PrP^Sc^ deposits in peripheral sites only, with PrP^Sc^ accumulations in the CMGC (ZYP24, ZYP37, both wild type genotypes) and also in the LRS of goat ZYP24.

Nevertheless, in goats with negative PrP^Sc^ test results in the brain stem a higher variability of genotypes was demonstrated with one goat combining two polymorphisms relative to the wild type genotype (ZYP39). Therefore reduced susceptibility for scrapie was confirmed for the goats of this study showing at least one PRNP polymorphism (Fisher’s exact test *p* = 0.023) with particularly goats showing polymorphisms at codon 146 being significantly more frequent TSE negative (Fisher’s exact test *p* = 0.016). These observations are in accordance with previous reports, clearly demonstrating a higher resistance of N/D146 and N/S146 PrP gene mutations to natural and experimental scrapie and even a complete resistance of homozygous polymorphisms of this type [12, 13, 53]. Additionally, cell-free conversion assays revealed an inhibitory potential of these genotypic variants, too [54]. It cannot be disregarded that the amount of goats examined in this study does not meet the requirements of a representative sample and that only six codons of the PRNP were considered, but still the results support a breeding strategy focussing on codon 146 to control and eradicate classical scrapie from Cyprus [53]. Whether there was any influence on TSE susceptibility due to the polymorphisms R151H and R154H cannot be ruled out in this study, though for the latter protective effects have already been described before [10, 12, 16, 18]. The M/M142 polymorphism was found in a negative goat 3 years of age. This is not surprising as mutations at codon 142 are associated with a lengthened incubation period in experimental inoculations and with increased resistance under natural conditions [15, 17, 18].

The present study did not reveal any indication that natural BSE occurs in goats of this part of Cyprus. Without exception all TSE positive brain samples of 25 goats including the ovine scrapie isolate (Stdl. 171) were characterized scrapie-like using the discriminatory FLI-test. A BSE case would be only indicated if the glycoform ratio for the diglycosylated form of the PrP^Sc^ is higher than 50%, the antibody binding ratio P4/L42 has a lower value than 0.4 and the molecular mass of the unglycosylated band is more than 0.5 kDa lower as compared to the internal scrapie control [49]. Therefore BSE was exclusively determined for the ovine, caprine and bovine BSE control samples. Eight caprine scrapie isolates revealed some discrepancies in the glycosylation pattern showing a diglycosylated form of the PrP^Sc^ with more than 50% of the overall signal which points to BSE-like characteristics. As all other parameters have clear scrapie-like attributes, these isolates were considered as classical scrapie, too. For scrapie in sheep it is well established that differences in biochemical and immunohistochemical properties are indicative for strain diversity in the natural host [49, 55–58]. More recently others reported molecular differences in goat isolates, too [42, 53, 59]. All eight goats are of the same wild type genotype and originate from similar or at least contact herds. Therefore an infection of these goats with the same strain cannot be excluded, but whether the biochemical pattern described here suggests similar strain diversity remain to be clarified by mouse bioassay [60].

In an additional approach four of the eight isolates with discordant biochemical properties and a representative sample from the other scrapie isolates as well as ovine and caprine BSE samples were analyzed by long-term PK digestion over a 48 h period. In doing so, no clear differences were seen among the caprine scrapie isolates but these were clearly less resistant against PK digestion than the caprine and ovine BSE isolates. The PK resistance of PrP^Sc^ was demonstrated to depend on the TSE form [57, 61–63] and recent studies even showed a differentiation of classical scrapie isolates in goats based on the sensitivity to PK digestion at the N-terminal cleavage site [59]. Further studies might show possible species and strain derived influences on the PK resistance.

Most of the scrapie positive tested goats exhibited widespread accumulations of PrP^Sc^ in samples of the CMGC and in the ENS as well as in samples of the LRS, most commonly in the retropharyngeal lymph node followed from accumulations in spleen, tonsils, lymphoid rectal tissue and the nictitating membrane. Similar distribution patterns of PrP^Sc^ were also reported in classical scrapie affected goats [42, 43, 46] and sheep [33, 35, 37, 51, 64], respectively. However, one of the scrapie positive goats (ZYP40) revealed besides a moderate accumulation of PrP^Sc^ in the obex region only severe PrP^Sc^ depositions in the rectal myenteric plexus and mild accumulations in the CMGC. Moreover, a restricted involvement of the LRS was noticed in this goat with mild PrP^Sc^ accumulations in the retropharyngeal lymph node, only. The goat was 4 years old and had a R154H polymorphism of the PRNP. In goats the R154H polymorphism was described to be associated with a prolonged incubation period and also with partially protective effects against classical scrapie [10, 12, 16]. Furthermore, a similar pattern was observed in Cyprus [53] and [58] reported influences of the I142M polymorphism on the PrP^Sc^ distribution pattern in samples of the caprine central nervous system, LRS and ENS. Thus, concerning goat ZYP40 gene based restriction of PrP^Sc^ distribution and the prolonged incubation period might be plausible, though our data were too limited for a proof. Alternatively this pattern could indicate a strain dependent effect. For example in susceptible sheep with a positive reaction in the brain stem a limited involvement of the LRS has also been shown in individual animals [51]. Further studies using mouse bioassays for strain typing purposes might clarify this question.

Moreover, in all scrapie positive goats and one scrapie negative goat (ZYP24) at least one sample was available for ante-mortem diagnosis (tonsil, RAMALT, nictitating membrane) and they always exhibited clear-cut PrP^Sc^ deposits. However it has to be emphasized that the comparably high frequency of detectable amounts of PrP^Sc^ in the RAMALT in our study is in contrast to the results reported by others, showing only a limited frequency of PrP^Sc^ detection in this tissue sample [42]. Additionally, one of the scrapie negative goats (ZG 37) showed a mild PrP^Sc^ accumulation in CMGC only and would have been missed by these methods, too. Although a false ante-mortem diagnostic might happen in individual cases, these diagnostic measures could be helpful to differentiate PrP^Sc^ positive goats from clinical suspects as shown by others in sheep [64–66].

A less easily accessible sample is the CMGC. It was one of the most frequent PrP^Sc^ positive samples, even positive in an otherwise negative goat. To the best of our knowledge such data was never described in scrapie affected goats before. However, [32] described similar findings in scrapie affected sheep indicating a similar early neuronal spread of PrP^Sc^ from the ENS via the CMGC to the central nervous system (CNS) in both species. Thus further similarities in caprine and ovine scrapie pathogenesis can be consolidated. However, discrepancies in the PrP^Sc^ distribution pattern between our results and others were seen concerning the involvement of the ENS in goat scrapie. In our hands most of the clinical affected animals showed a clear accumulation in neurons and satellite cells of the rectal plexus, a region which is known for ovine classical scrapie to be involved at later stages of the disease [32]. In contrast goats from the UK classical scrapie outbreak [43] showed an only inconsistent accumulation of PrP^Sc^ in the ileal ENS, a region which is highly involved in early ovine TSE pathogenesis at least in the highly scrapie susceptible VRQ/VRQ sheep [32]. Additionally in contrast to earlier studies where PrP^Sc^ deposits were detected in the kidney of scrapie affected goats [46] and sheep [39] and also in the ovine udder [67], PrP^Sc^ was not found in the kidneys and in the udder of the goats of this study. To which extent these patterns are due to the species and genotype of the host, strain diversity and/or the progress of the disease still needed to be clarified. However, in three out of 15 goats mild PrP^Sc^ accumulations were found in the fetal (trophoblasts) and maternal parts (epithelial cells) of the placental cotyledons. This is consistent with other studies in scrapie positive goats [68] and sheep [69]. All three she-goats showed wide-spread PrP^Sc^ deposits in samples of the LRS and in the obex samples implicating that PrP^Sc^ is accumulating in placental tissue at a late stage of scrapie pathogenesis. In sheep, PrP^Sc^ accumulation is assumingly highly influenced by the stage of pregnancy and the fetal PRNP genotype [69, 70]. These two parameters were not captured in this study preventing to draw further conclusions in this item. Nevertheless, it would be of high interest to find out whether placental PrP^Sc^ distribution correlates with the state of pathogenesis, to know the influence which is taken by the gestation period and the impact of maternal and fetal PRNP genotype in naturally affected goats together. Moreover, the importance of caprine placenta for scrapie transmission remains to be further clarified.

In summary, a biochemical and immunohistochemical characterization of natural goat TSE cases from different Cyprus herds was performed. The PrP^Sc^ was detected in the brain stem of 25 goats and 17 goats provided a negative result. Two of the latter goats exhibited PrP^Sc^ deposits in peripheral tissues only. The PrP^Sc^ negative goats with assumingly reduced scrapie susceptibility were associated with polymorphisms at codon 146. The discriminatory FLI-test allowed to proof, that all 25 positive brain stem isolates showed clear scrapie-like characteristics. However, eight isolates revealed different glycosylation patterns, which might be indicative for the existence of different scrapie strains. To our surprise, goat scrapie isolates showed lower resistance to long term PK digestion than caprine and ovine BSE samples. This fact was very surprising as ovine scrapie was demonstrated to be more resistant to long term PK digestion than BSE from sheep and cattle. However, no differences were seen between the goat isolates. Furthermore, in most goats, PrP^Sc^ accumulations were widely distributed within ENS, the LRS and the CMGC. Even samples which are accessible for ante mortem diagnostics (tonsils, RAMALT, third eye lid) were frequently tested PrP^Sc^ positive. However, it must be noted, that one goat with a R/H154 polymorphism showed an only minor involvement of the LRS. Further, the present study confirms the presence of PrP^Sc^ in trophoblasts and epithelial cells of cotelydons from scrapie-affected goats.

## Abbreviations

TSE: transmissible spongiform encephalopathy
BSE: bovine spongiform encephalopathy
PrP^c^/PrP^Sc^: cellular/pathological prion protein
PRNP: prion protein gene
CMGC: celiac and mesenteric ganglion complex
LRS: lymphoreticular system
ENS: enteric nervous system
RAMALT: rectoanal mucosa-associated lymphoid tissue
